# Involvement of Hydrogen Peroxide in Safingol-Induced Endonuclease G-Mediated Apoptosis of Squamous Cell Carcinoma Cells

**DOI:** 10.3390/ijms15022660

**Published:** 2014-02-17

**Authors:** Masakazu Hamada, Ken Wakabayashi, Atsushi Masui, Soichi Iwai, Tomoaki Imai, Yoshiaki Yura

**Affiliations:** Department of Oral and Maxillofacial Surgery, Osaka University Graduate School of Dentistry, 1-8 Yamadaoka, Suita, Osaka 565-0871, Japan; E-Mails: dental_ken@yahoo.co.jp (K.W.); a-masui@dent.osaka-u.ac.jp (A.M.); s-iwai@dent.osaka-u.ac.jp (S.I.); hsc12@hotmail.com (T.I.); yura@dent.osaka-u.ac.jp (Y.Y.)

**Keywords:** safingol, hydrogen peroxide, apoptosis, endonuclease G

## Abstract

Safingol, a L-threo-dihydrosphingosine, induced the nuclear translocation of a mitochondrial apoptogenic mediator—endonuclease G (endo G)—and apoptosis of human oral squamous cell carcinoma (SCC) cells. Upstream mediators remain largely unknown. The levels of hydrogen peroxide (H_2_O_2_) in cultured oral SCC cells were measured. Treatment with safingol increased intracellular H_2_O_2_ levels but not extracellular H_2_O_2_ levels, indicating the production of H_2_O_2_. The cell killing effect of safingol and H_2_O_2_ was diminished in the presence of reactive oxygen species (ROS) scavenger *N*-acetyl-L-cysteine (NAC). Dual staining of cells with annexin V and propidium iodide (PI) revealed that apoptotic cell death occurred by treatment with H_2_O_2_ and safingol. The number of apoptotic cells was reduced in the presence of NAC. In untreated cells, endo G distributed in the cytoplasm and an association of endo G with mitochondria was observed. After treatment with H_2_O_2_ and safingol, endo G was distributed to the nucleus and cytoplasm, indicating the nuclear translocation of the mitochondrial factor. NAC prevented the increase of apoptotic cells and the translocation of endo G. Knock down of endo G diminished the cell killing effect of H_2_O_2_ and safingol. These results suggest that H_2_O_2_ is involved in the endo G-mediated apoptosis of oral SCC cells by safingol.

## 1. Introduction

Apoptosis, the best-described type of programmed cell death, is characterized by cell membrane blebbing, a reduction in cellular volume, the activation of caspases, chromatin condensation and nuclear fragmentation [1,2]. Internucleosomal DNA fragmentation is a hallmark of the apoptotic process and at least two endonucleases, caspase-activated DNase (CAD) and endonuclease G (endo G), are thought to be important for mammalian DNA fragmentation during apoptosis [3,4]. The best-characterized major enzyme for DNA fragmentation is the CAD that forms an inactive heterodimer with inhibitor of CAD (ICAD). Following apoptotic signaling, ICAD is proteolyzed by caspase-3 causing the dissociation of the CAD/ICAD heterodimer and releasing CAD, which then moves from the cytosol to the nucleus. Endo G is an endonuclease that is released from the mitochondrial intermembrane space and translocates to the cell nucleus to induce DNA fragmentation in a caspase-independent manner [4–6].

Safingol, a L-threo-dihydrosphingosine, is a synthetic lipid and functions by targeting the lipid-binding regulatory domain of protein kinase C (PKC) [7,8]. In previous studies, safingol was used as a PKCα-selective inhibitor and antitumor activity was demonstrated [8–12]. The cytotoxic effect of safingol was also attributed to the inhibition of sphingosine kinase 1, thus preventing the formation of sphingosine-1-phosphate, which is involved in cell proliferation, invasion and angiogenesis [13–15]. Safingol is currently under a phase I clinical trial in combination with cisplatin for the treatment of advanced solid tumors [16]. Our previous studies indicated that safingol induced apoptosis of oral squamous cell carcinoma (SCC) cells, accompanied by the nuclear translocation of endo G from mitochondria in a caspase 3-independet manner, using DNA fragmentation assay, flow cytometric analysis and immunostaining [17], but upstream mediators remain largely unknown.

Oxidative stress has been implicated in a number of physiological and pathological processes, including cancer, ischemic injury, neurodegenerative diseases, chronic inflammation, type II diabetes and arteriosclerosis [18]. Reactive oxygen species (ROS) are recognized as chemical mediators in deciding the fate of cells, depending on the extent of oxidative damage. In the present study, we investigated the possible involvement of H_2_O_2_ as a ROS in endo G-mediated apoptosis of oral SCC cells treated with safingol.

## 2. Results

### 2.1. Production of ROS in SCC Cells by Treatment with Safingol

**Figure 1.**
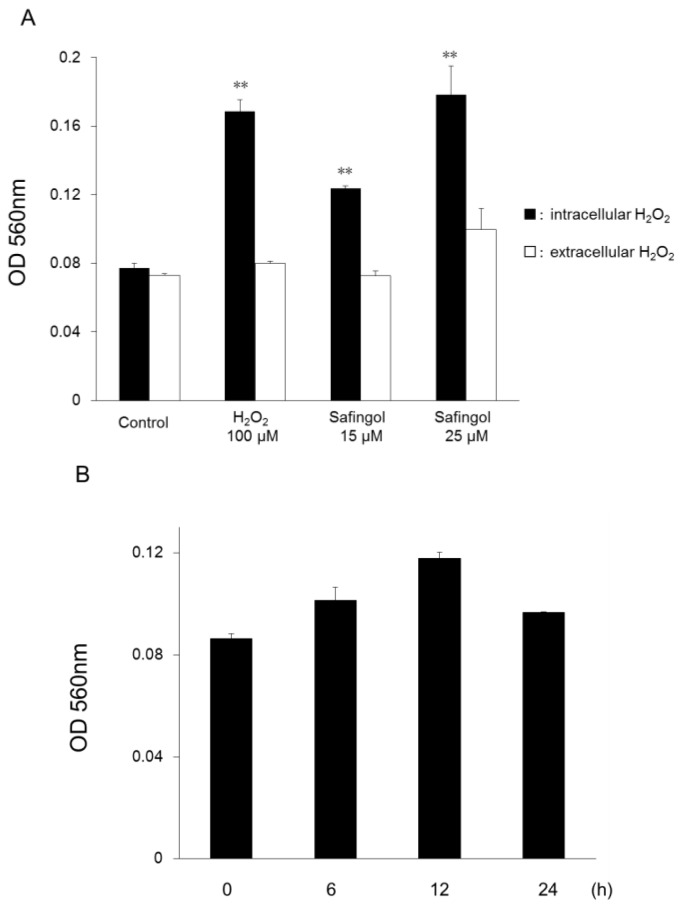
Production of ROS in SCC cells treated with safingol. SAS cells were treated with H_2_O_2_ or safingol, and extracellular and intracellular ROS levels were determined 12 h later (**A**); SAS cells were treated with 15 μM safingol, and intracellular ROS levels were determined 0, 6, 12, 24 h later (**B**). The data represent the mean ± SD of three determinations. ** *p* < 0.01 *vs.* control.

SAS cells were incubated with hydrogen peroxide (H_2_O_2_) or safingol, and extracellular and intracellular levels of H_2_O_2_ were measured using an assay kit for measuring H_2_O_2_ concentration [19,20] 12 h later. After treatment with 100 μM H_2_O_2_, the intracellular H_2_O_2_ concentration increased, but the extracellular H_2_O_2_ concentration did not (Figure 1A). When SAS cells were treated with safingol at 15 or 25 μM, the H_2_O_2_ levels in the cells also increased. The difference between the treated cells and untreated control was significant. The level of H_2_O_2_ in the medium of the cells treated with H_2_O_2_ or safingol was not altered. When SAS cells were treated with 15 μM safingol for 6, 12, and 24 h, the intracellular H_2_O_2_ concentration increased and reached a max level of 12 h.

### 2.2. Induction of Cell Death by H_2_O_2_ and Safingol

**Figure 2.**
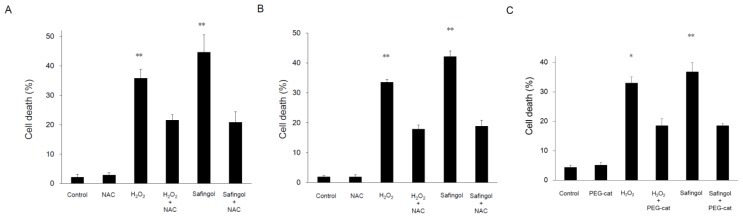
Induction of cell death by H_2_O_2_ and safingol. SAS (**A**) and HSC-3 (**B**) cells were treated with 100 μM H_2_O_2_ or 15 μM safingol alone. Alternatively, they were treated with H_2_O_2_ or safingol in the presence of the ROS scavenger NAC for 12 h. SAS cells were treated with 100 μM H_2_O_2_ or 15 μM safingol alone. Alternatively, they were pretreated with 500U PEG catalase (PEG-cat) for 2 h and then they were treated with H_2_O_2_ or safingol for 12 h (**C**). Thereafter, the cells were stained with trypan blue. The percentages of dead cells were calculated. The data represent the mean ± SD of three determinations. * *p* < 0.05, ** *p* < 0.01 *vs.* treated group with NAC or PEG-cat.

**Figure 3.**
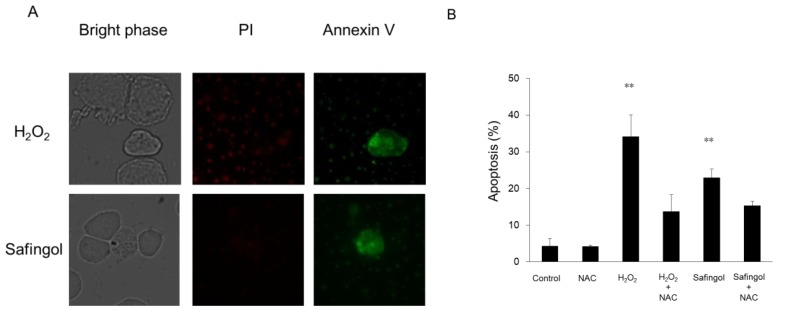
Induction of apoptotic cells death by H_2_O_2_ and safingol. SAS cells were treated with 100 μM H_2_O_2_ or 15 μM safingol alone (**A**). Alternatively, they were also treated with H_2_O_2_ or safingol in the presence of the ROS scavenger NAC for 12 h. Thereafter, the cells were stained with annexin V and PI. The percentages of apoptotic cells stained with annexin V alone were calculated (**B**). The data represent the mean ± SD of three determinations. ** *p* < 0.01 *vs.* treated group with NAC.

Cell death was examined using the trypan blue dye exclusion test. When SAS cells were treated with 100 μM H_2_O_2_ for 12 h, the proportion of dead cells increased to 36%, though this increase was diminished in the presence of a ROS scavenger, *N*-acetyl-L-cysteine (NAC) [21,22], with 22% of cells nonviable (Figure 2A). When cells were treated with 15 μM safingol for 12 h, 45% were found to be nonviable. This value was reduced to 21% by NAC. When another oral SCC cell line HSC-3 was used, H_2_O_2_ and safingol decreased the proportion of viable cells in a similar manner as observed in SAS cells. The suppressive effect was blunted by NAC (Figure 2B). When 500U PEG catalase (PEG-cat) was used to delete H_2_O_2_ production by safingol, the cell killing effect of safingol was decreased (Figure 2C). SAS cells were treated with H_2_O_2_ or safingol and dual staining with annexin V and propidium iodide (PI) was performed. Cells stained with annexin V alone were considered to be apoptotic cells. The percentage of apoptotic cells was also increased by treatment with H_2_O_2_ and safingol, up to 34% and 23%, respectively (Figure 3). These values decreased to 14% and 15% in the presence of NAC.

### 2.3. Effect of Endo G Small Interfering RNA (siRNA) on the Cell Death Caused by H_2_O_2_ and Safingol

**Figure 4.**
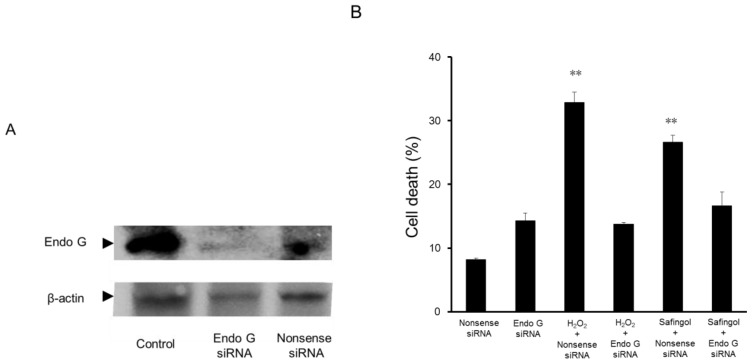
Effect of endo G siRNA transfection on cell viability. (**A**) SAS cells were transfected with endo G siRNA or nonsense siRNA and cultured for 24 h. They were subjected to an immunoblot analysis. At least three determinations were performed. A representative result is shown; (**B**) Endo G siRNA- or nonsense siRNA-transfected SAS cells were treated with 300 μM H_2_O_2_ or 15 μM safingol for 12 h and subjected to a trypan blue dye exclusion test. The data represent the mean ± SD of three determinations. ** *p* < 0.01 *vs.* treated group with endo G siRNA transfection.

Previously, we reported that safingol induced the translocation of endo G from mitochondria to the nucleus and induced apoptosis [17]. In the present study, the effect of siRNA on cell viability was examined. SAS cells were transfected with endo G siRNA and subjected to immunoblotting. The expression of endo G was downregulated by this treatment, whereas it was maintained after the transfection of nonsense siRNA (Figure 4A).

Treatment with 300 μM H_2_O_2_ and 15 μM safingol increased the percentage of dead cells to 33% and 27%, respectively, in the cultures transfected with nonsense siRNA. In endo G siRNA-transfected cells, these values decreased to 14% and 17%, respectively, indicating the involvement of endo G in the H_2_O_2_- and safingol-induced cell death (Figure 4B).

### 2.4. Translocation of Endo G by H_2_O_2_ and Safingol

**Figure 5.**
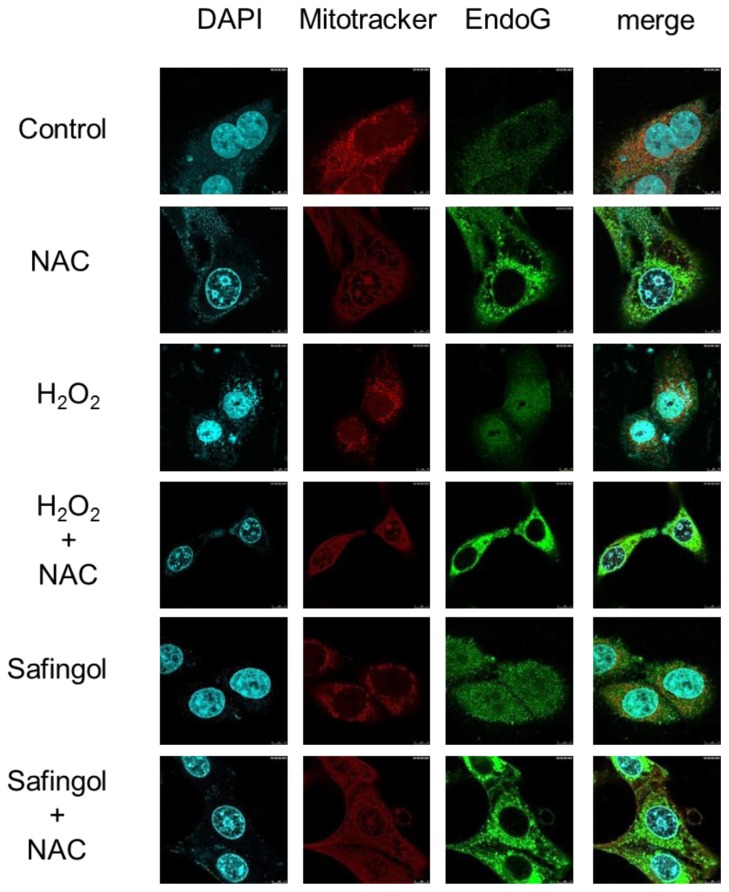
Translocation of endo G by H_2_O_2_ and safingol. SAS cells were treated with 100 μM H_2_O_2_ or 15 μM safingol in the presence or absence of NAC for 12 h. They were subjected to immunofluorescent staining using DAPI, antibody against endo G and Mitotracker Red CMXRos DAPI. Untreated cells were also stained. The imaging of each staining was merged. At least three measurements were performed. A representative result is shown.

The effect of H_2_O_2_ and safingol on the localization of mitochondria and the expression of endo G were examined using immunofluoresent antibody staining. In untreated SAS cells, the mitochondria were filamentous with a tubular appearance and often interconnected forming a network. Most cytoplasmic staining of endo G was co-localized with mitochondria, and specific nuclear staining was not observed (Figure 5). After treatment with 100 μM H_2_O_2_, the localization of mitochondria was unchanged, but endo G showed diffuse distribution. Inconsistent with the results for 4′,6-diamidino-2-phenylindole (DAPI) staining, nuclear staining of endo G was observed. When cells were treated with H_2_O_2_ in the presence of NAC, the endo G was confined to the cytoplasm and nuclear localization was not observed. Safingol also induced nuclear staining of endo G, which was completely blocked by NAC.

## 3. Discussion

We had shown that safingol could induce cell death with characteristics of apoptosis at a concentration of 25 μM in a caspase three-independent manner [23]. At 10 μM, however, though a proportion of cells detached, they reattached on the plate after prolonged incubation. The cell killing effect of safingol was marginal at this concentration [17,23]. On the other hand, safingol was reported to exert an inhibitory effect on sphingosine kinase 1 at concentrations below 10 μM [14,16]. Since our previous study was undertaken at higher concentrations, safingol would affect sphingosine kinase 1 as well as PKC, to induce apoptosis of oral SCC cells.

Activation of apoptosis is associated with the generation of ROS [24]. Indeed, some anticancer drugs induce production of ROS during apoptosis [25–27]. Mizutani *et al.* [27] reported that the critical apoptotic trigger of doxorubicin, a topoisomerase II inhibitor, was oxidative DNA damage from doxorubicin-induced H_2_O_2_ production and that the oxidative damage caused the indirect generation of H_2_O_2_ through poly (ADP-ribose) polymerase (PARP) and nicotinamide adenine dinucleotide phosphate (NADPH) oxidase activation, leading to an increase in mitochondrial membrane permeability. To determine the possible involvement of ROS in safingol-induced cell death in oral SCC cells, we examined the levels of intracellular and extracellular ROS and found that safingol as well as H_2_O_2_ increased intracellular H_2_O_2_ level. When cell death was estimated using the trypan blue dye exclusion test, H_2_O_2_ and safingol induced cell death and the killing effect was efficiently blocked by a ROS scavenger, NAC, in SAS and HSC-3 cells. Apoptotic cells stained with annexin V alone were also reduced by NAC in SAS cells. The difference observed in trypan blue and annexin V staining especially in safingol-treated cells may represent apoptotic cells with necrotic degradation, because apoptotic cells looks like necrotic cells at an advanced stage. Thus, it can be stated that safingol produces ROS including H_2_O_2_, which is responsible for the induction of apoptotic cell death in oral SCC cells. Mitochondria itself produces ROS, but H_2_O_2_ that was added to the cell culture induced apoptosis in the present study. Cytoplasmic H_2_O_2_ must be the inducer of release of apoptogenic mitochondrial factors. Ling *et al.* [28] indicated that safingol caused time-and concentration-dependent production of ROS in MDA-MB-231 and HT-29 cells, suggesting ROS to be a mediator of safingol-induced cancer cell death. They cultured the cells for 48 h at 10 μM and found necrosis and autophagy, but they did not examine DNA fragmentation observed in apoptosis. Since safingol at 10 μM did not induce cell death in most SAS cells, we have not examined the role of autophagy as observed in MDA-MB-231 and HT-29 cells.

Intrinsic apoptosis is critically dependent on mitochondrial outer membrane permeabilization, which results in the release of mitochondrial intermembrane space proteins, such as cytochrome c, and endo G [29,30]. In the present study, we found that the effect of H_2_O_2_ and safingol was blocked by the downregulation of endo G expression, indicating that endo G is required for the cell death by the treatment. We also found that H_2_O_2_ as well as safingol induced translocation of endo G to the nucleus. The expression of endo G was not necessarily correlated with the intensity of mitochondrial staining, but was present in the cytoplasm of untreated oral SCC cells, representing the synthesis of the mitochondrial protein in the cytoplasm. After treatment with H_2_O_2_ or safingol, the nuclear accumulation of endo G occurred, but cytoplasmic staining was preserved. Apoptotic signal would stimulate the release of endo G from mitochondria to the cytoplasm and then the endo G comcomitantly with the preexisting cytoplasmic endo G move to the nucleus for DNA fragmentaion. It should be also stated that NAC clearly blocked the alteration of endo G staining by H_2_O_2_ and safingol. In a neuronal system, Higgins *et al.* [31] found that oxidative stress triggered neuronal caspase-independent cell death and the translocation of endo G. Treatment caused the redistribution from mitochondria of both endo G and cytochrome c. Kim *et al.* [32] treated head and neck cancer cells with cisplatin and found mitochondrial outer membrane permeabilization, the nuclear translocation of endo G and apoptosis. Together, we firstly suggest that the expression and translocation of endo G are required for the inducation of cell death of oral SCC cells by safingol and that H_2_O_2_ is one of the upstream factors in this event.

## 4. Experimental Section

### 4.1. Cell Culture

The human oral SCC cell line SAS and HSC-3 were obtained from the Japanese Collection of Research Bioresources (Tokyo, Japan). Cells were cultured in Dulbecco’s modified Eagle’s medium (DMEM) supplemented with 5% fetal bovine serum, 2 mM l-glutamine, 100 μg/mL penicillin and 100 μg/mL streptomycin and grown in an incubator at 37 °C in a humidified atmosphere with 5% CO_2_.

### 4.2. Reagents

Safingol was obtained from Calbiochem-Novabiochem (San Diego, CA, USA). H_2_O_2_ and NAC were obtained from Wako (Osaka, Japan) and PEG-cat was obtained from Sigma (St. Louis, MO, USA).

### 4.3. Measurement of H_2_O_2_

The concentration of H_2_O_2_ was determined using a colorimetric assay. Cells were plated in 48-well plates at a density of 2 × 10^4^ cells/well and treated with H_2_O_2_ or safingol for 12 h. The supernatant of SAS cells was harvested as an extracellular sample. Cells were dissociated with an ethylenediaminetetraacetic acid (EDTA)-trypsin solution, subjected to three cycles of freezing and thawing and used as an intracellular sample for the H_2_O_2_ assay [19,20]. Ten microliters of sample was mixed with 100 μL of Bioxytech H_2_O_2_-560 (OXIS International, Portland, OR, USA) and incubated for 30 min at room temperature. Measurements were made using a Benchmark plus microplate spectrophotometer (Bio-Ras Laboratories, Hercules, CA, USA) at a wavelength of 560 nm.

### 4.4. Trypan Blue Staining

Cell viability was determined by the trypan blue dye exclusion test. Cells were plated in 6-well plates at a density of 1 × 10^6^ cells/well, cultured for 24 h and treated with 100 μM H_2_O_2_ or 15 μM safingol for 12 h. They were dissociated by the EDTA-trypsin solution, and cells were centrifuged suspended in phosphate-buffered saline (PBS) without Ca^2+^ and Mg^2+^. The pellets were then mixed with an equal volume of PBS without Ca^2+^ and Mg^2+^ containing 0.4% trypan blue and observed with a microscope. We counted the numbers of stained and unstained cells. Results were compared to those for the untreated controls and a percentage was calculated.

### 4.5. Annexin V and PI Staining

To identify apoptotic cells, annexin V and PI staining was performed using Vybrant Apoptosis Assay Kit (Life Technologies Corporation, Carlsbad, CA, USA) according to the manufacturer’s directions. After treatment with H_2_O_2_ or safingol, floating cells were harvested with medium and attached cells were dissociated with EDTA-trypsin solution. These cells were collected by centrifugation at 1000 rpm for 5 min. The cell pellets were suspended in 100 μL binding buffer (10 mM HEPES, 140 mM NaCl, 2.5 mM CaCl_2_, pH 7.4) and incubated with 5 μL FITC Annexin V and 1 μL of a propidium iodide (100 μg/mL) solution for 15 min at room temperature. Staining for annexin V and propidium iodide was observed under a fluorescence microscope (Microphoto FXA; Nikon, Tokyo, Japan). The percentages of apoptotic cells stained with annexin V alone were calculated. At least 3 samples and 1000 cells were counted for determination of the percentage of apoptotic cells.

### 4.6. Immunoblot Analysis

Cells were washed in PBS and lysed in a buffer containing 20 mM Tris-HCl (pH 7.4), 0.1% SDS, 1% TritonX-100, 1% sodium deoxycholate and protease inhibitor cocktail. After sonication on ice and subsequent centrifugation at 15,000× *g* for 10 min at 4 °C, the supernatant was collected and the protein concentration was determined using a Protein Assay Kit (Bio-Rad, Hercules, CA, USA). Sample protein (15 μg) was electrophoresed through a polyacrylamide gel and transferred to a polyvinylidene fluoride membrane (Millipore, Bedford, MA, USA) by electroblotting. The membrane was probed with antibodies and antibody-binding was detected using an enhanced chemiluminescence kit (GE Healthcare, Amersham, Buckinghamshire, UK) according to the manufacturer’s instructions. The antibodies used were a rabbit polyclonal antibody against endo G (Sigma, St. Louis, MO, USA), and β-actin (Sigma, St. Louis, MO, USA). The secondary antibodies used were horseradish peroxidase-conjugated anti-rabbit IgG (Cell Signaling Technology, Beverly, MA, USA) and peroxidase-conjugated anti-mouse IgG (Sigma, St. Louis, MO, USA).

### 4.7. siRNA Transfection

Chemically synthetic siRNA against endo G and AllStars negative control siRNA (nonsense siRNA) were purchased from Qiagen (Valencia, CA, USA). The target sequence of the siRNA for endo G was 5′-AAAUGCCUGGAACAACCUUGA-3′. Cells were plated in 6-well plates at a density of 1 × 10^5^ cells/well, cultured for 24 h, and transfected with 40 nM endo G siRNA or nonsense siRNA using Lipofectamine 2000 (Invitrogen, Carlsbad, CA, USA) according to the manufacturer’s directions. The medium was replaced with DMEM after 3 h and cells were used for experiments at 24 h after transfection.

### 4.8. Confocal Laser Microscopic Analysis

Cells were treated with H_2_O_2_ or safingol for 12 h. Thereafter, they were fixed with 2% paraformaldehyde, permealized with 0.1% Triton X-100 in PBS and incubated with 25 nM Mitotracker Red CMXRos (Molecular Probes, Eugene, OR, USA) at 37 °C for 45 min. They were fixed in 4% paraformaldehyde phosphate buffer solution (WAKO, Osaka, Japan), permealized with 0.1% Triton X-100 in PBS and incubated with a rabbit polyclonal antibody against endo G (Sigma, St. Louis, MO, USA) diluted 1:200 in PBS for 1 h at room temperature. After washing, the cells were incubated with Alexa Fluor 488 goat antirabbit antibody (Life Technologies Corporation, Carlsbad, CA, USA) diluted 1:500 in PBS for 1 h. After washing, coverslips were mounted onto microslides using a ProLong Gold Antifade Reagent with DAPI (Life Technologies Corporation, Carlsbad, CA, USA). The slides were analyzed under a confocal laser-scanning microscope Leica TCS SP8 (Leica Microsystems, Mannheim, Germany).

### 4.9. Statistical Analysis

The statistical analysis was performed using a Student’s *t* test with Microsoft Excel (Windows vista, Microsoft, Redmond, WA, USA). The results were expressed as the mean ± SD. The differences were considered significant at *p* < 0.05.

## 5. Conclusions

Safingol mimics H_2_O_2_ in the ability to induce the death of oral SCC cells. H_2_O_2_ as a ROS is suggested to act as upstream mediators to induce translocation of endo G which can directly contribute to the cleavage of nuclear DNA.

## References

[1] Kroemer G., Galluzzi L., Vandenabeele P., Abrams J., Alnemri E.S., Baehrecke E.H., Blagosklonny M.V., El-Deiry W.S., Golstein P., Green D.R. (2009). Classification of cell death: recommendations of the Nomenclature Committee on Cell Death 2009. Cell. Death Differ.

[2] Ghobrial I.M., Witzig T.E., Adjei A.A. (2005). Targeting apoptosis pathways in cancer therapy. CA: A Cancer J. Clin.

[3] Sakahira H., Enari M., Nagata S. (1998). Cleavage of CAD inhibitor in CAD activation and DNA degradation during apoptosis. Nature.

[4] Li L.Y., Luo X., Wang X. (2001). Endonuclease G is an apoptotic DNase when released from mitochondria. Nature.

[5] Lorenzo H.K., Susin S.A. (2004). Mitochondrial effectors in caspase-independent cell death. FEBS Lett.

[6] Li J., Zhou J., Li Y., Qin D., Li P. (2010). Mitochondrial fission controls DNA fragmentation by regulating endonuclease G. Free Radic. Biol. Med.

[7] Hannun Y.A., Loomis C.R., Merrill A.H., Bell R.M. (1986). Sphingosine inhibition of protein kinase C activity and of phorbol dibutyrate binding *in vitro* and in human platelets. J. Biol. Chem.

[8] Kedderis L.B., Bozigian H.P., Kleeman J.M., Hall R.L., Palmer T.E., Harrison S.D., Susick R.L. (1995). Toxicity of the protein kinase C inhibitor safingol administered alone and in combination with chemotherapeutic agents. Fundam. Appl. Toxicol.

[9] Schwartz G.K., Haimovitz-Friedman A., Dhupar S.K., Ehleiter D., Maslak P., Lai L., Loganzo F., Kelsen D.P., Fuks Z., Albino A.P. (1995). Potentiation of apoptosis by treatment with the protein kinase *C*-specific inhibitor safingol in mitomycin *C*-treated gastric cancer cells. J. Natl. Cancer Inst.

[10] Hoffmann T.K., Leenen K., Hafner D., Balz V., Gerharz C.D., Grund A., Ballo H., Hauser U., Bier H. (2002). Antitumor activity of protein kinase C inhibitors and cisplatin in human head and neck squamous cell carcinoma lines. Anticancer Drugs.

[11] Choe Y., Jung H., Khang I., Kim K. (2003). Selective roles of protein kinase C isoforms on cell motility of GT1 immortalized hypothalamic neurones. J. Neuroendocrinol.

[12] Uemura K., Aki T., Yamaguchi K., Yoshida K. (2003). Protein kinase C-epsilon protects PC12 cells against methamphetamine-induced death: Possible involvement of suppression of glutamate receptor. Life Sci.

[13] Buehrer B.M., Bell R.M. (1993). Sphingosine kinase: Properties and cellular functions. Adv. Lipid Res.

[14] Olivera A., Kohama T., Tu Z., Milstien S., Spiegel S. (1998). Purification and characterization of rat kidney sphingosine kinase. J. Biol. Chem.

[15] Pyne N.J., Pyne S. (2010). Sphingosine 1-phosphate and cancer. Nat. Rev. Cancer.

[16] Dickson M.A., Carvajal R.D., Merrill A.H., Gonen M., Cane L.M., Schwartz G.K. (2011). A phase I clinical trial of safingol in combination with cisplatin in advanced solid tumors. Clin. Cancer Res.

[17] Hamada M., Sumi T., Iwai S., Nakazawa M., Yura Y. (2006). Induction of endonuclease G-mediated apopotosis in human oral squamous cell carcinoma cells by protein kinase C inhibitor safingol. Apoptosis.

[18] Azad M.B., Chen Y., Gibson S.B. (2009). Regulation of autophagy by reactive oxygen species (ROS): Implications for cancer progression and treatment. Antioxid Redox. Signal.

[19] Jiang Z.Y., Woollard A.C., Wolff S.P. (1990). Hydrogen peroxide production during experimental protein glycation. FEBS Lett.

[20] Jiang Z.Y., Hunt J.V., Wolff S.P. (1992). Ferrous ion oxidation in the presence of xylenol orange for detection of lipid hydroperoxide in low density lipoprotein. Anal. Biochem.

[21] Zafarullah M., Li W.Q., Sylvester J., Ahmad M. (2003). Molecular mechanisms of *N*-acetylcysteine actions. Cell. Mol. Life Sci.

[22] Aruoma O.I., Halliwell B., Hoey B.M., Butler J. (1989). The antioxidant action of *N*-acetylcysteine: Its reaction with hydrogen peroxide, hydroxyl radical, superoxide, and hypochlorous acid. Free Radic. Biol. Med.

[23] Noda T., Iwai S., Hamada M., Fujita Y., Yura Y. (2009). Induction of apoptosis of detached oral squamous cell carcinoma cells by safingol. Possible role of Bim, focal adhesion kinase and endonuclease G. Apoptosis.

[24] Cai J., Jones D.P. (1998). Superoxide in apoptosis. Mitochondrial generation triggered by cytochrome c loss. J. Biol. Chem.

[25] Gewirtz D.A. (1999). A critical evaluation of the mechanisms of action proposed for the antitumor effects of the anthracycline antibiotics adriamycin and daunorubicin. Biochem. Pharmacol.

[26] Varbiro G., Veres B., Gallyas F., Sumegi B. (2001). Direct effect of taxol on free radical formation and mitochondrial permeability transition. Free Radic. Biol. Med.

[27] Mizutani H., Tada-Oikawa S., Hiraku Y., Kojima M., Kawanishi S. (2005). Mechanism of apoptosis induced by doxorubicin through the generation of hydrogen peroxide. Life Sci.

[28] Ling L.U., Tan K.B., Lin H., Chiu G.N. (2011). The role of reactive oxygen species and autophagy in safingol-induced cell death. Cell Death Dis.

[29] Zamzami N., Kroemer G. (2004). Methods to measure membrane potential and permeability transition in the mitochondria during apoptosis. Methods Mol. Biol.

[30] Jourdain A., Martinou J.C. (2009). Mitochondrial outer-membrane permeabilization and remodelling in apoptosis. Int. J. Biochem. Cell Biol.

[31] Higgins G.C., Beart P.M., Nagley P. (2009). Oxidative stress triggers neuronal caspase-independent death: endonuclease G involvement in programmed cell death-type III. Cell. Mol. Life Sci.

[32] Kim J.S., Lee J.H., Jeong W.W., Choi D.H., Cha H.J., Kim D.H., Kwon J.K., Park S.E., Park J.H., Cho H.R. (2008). Reactive oxygen species-dependent EndoG release mediates cisplatin-induced caspase-independent apoptosis in human head and neck squamous carcinoma cells. Int. J. Cancer.

